# Improved Dissolution and Pharmacokinetics of Abiraterone through KinetiSol^®^ Enabled Amorphous Solid Dispersions

**DOI:** 10.3390/pharmaceutics12040357

**Published:** 2020-04-14

**Authors:** Urvi Gala, Dave Miller, Robert O. Williams

**Affiliations:** 1 Molecular Pharmaceutics and Drug Delivery Division, College of Pharmacy, The University of Texas at Austin, 2409 University Avenue, Austin, TX 78712, USA; urvi.gala@utexas.edu; 2 DisperSol Technologies LLC., 111 W. Cooperative Way, Building 3, Suite 300, Georgetown, TX 78626, USA; dave.miller@dispersoltech.com

**Keywords:** abiraterone, dissolution, pharmacokinetics, amorphous solid dispersions, KinetiSol^®^ technology

## Abstract

Abiraterone is a poorly water-soluble drug. It has a high melting point and limited solubility in organic solvents, making it difficult to formulate as an amorphous solid dispersion (ASD) with conventional technologies. KinetiSol^®^ is a high-energy, fusion-based, solvent-free technology that can produce ASDs. The aim of this study was to evaluate the application of KinetiSol to make abiraterone ASDs. We developed binary KinetiSol ASDs (KSDs) using both polymers and oligomers. For the first time, we reported that KinetiSol can process hydroxypropyl-β-cyclodextrin (HPBCD), a low molecular-weight oligomer. Upon X-ray diffractometry and modulated differential scanning calorimetry analysis, we found the KSDs to be amorphous. In vitro dissolution analysis revealed that maximum abiraterone dissolution enhancement was achieved using a HPBCD binary KSD. However, the KSD showed significant abiraterone precipitation in fasted state simulated intestinal fluid (FaSSIF) media. Hence, hypromellose acetate succinate (HPMCAS126G) was selected as an abiraterone precipitation inhibitor and an optimized ternary KSD was developed. A pharmacokinetic study revealed that HPBCD based binary and ternary KSDs enhanced abiraterone bioavailability by 12.4-fold and 13.8-fold, respectively, compared to a generic abiraterone acetate tablet. Thus, this study is the first to demonstrate the successful production of an abiraterone ASD that exhibited enhanced dissolution and bioavailability.

## 1. Introduction

**Figure 1:**
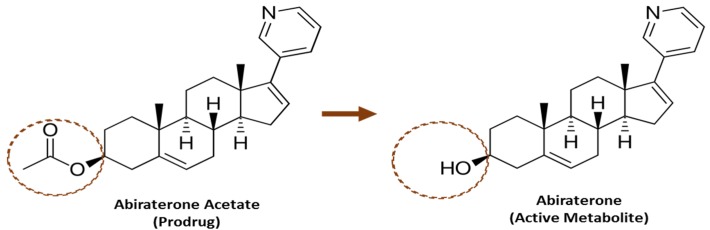
Structure of Abiraterone Acetate and Abiraterone.

Abiraterone acetate is approved for the treatment of metastatic castration-resistant prostate cancer (mCRPC) and metastatic high-risk castration-sensitive prostate cancer (mHCSPC) [1]. Abiraterone acetate is a prodrug that is converted predominantly pre-systemically to its active metabolite abiraterone (see Figure 1) via esterase-catalyzed hydrolysis [2]. Abiraterone is a potent and selective irreversible inhibitor of the enzyme CYP17A1, which is required for androgen biosynthesis [3,4]. It acts primarily by decreasing androgen production in the testicular, adrenal, and prostatic tumor tissues, thereby leading to slower disease progression [3,5].

Abiraterone has a water solubility of 3.05 µg/mL. It has low permeability and is considered as a BCS (Biopharmaceutical Classification System) Class IV compound [6]. Abiraterone also has poor solubility in biorelevant media such as FaHIF (fasted-state human intestinal fluid) and FaSSIF (fasted-state simulated intestinal fluid) [2]. Thus, abiraterone demonstrates low and variable oral bioavailability without an enabling formulation. Moreover, the melting point of abiraterone is high at 227.85 °C and it is also poorly soluble in most organic solvents [6]. Because of these physicochemical properties, it is exceptionally challenging to develop an abiraterone formulation with optimal stability and bioavailability. On the other hand, abiraterone acetate has a slightly higher solubility than abiraterone in biorelevant media such as FaHIF and FaSSIF [2]. Hence, due to the challenges associated with formulating abiraterone as is, it was formulated into a tablet dosage form called Zytiga^®^, which contains abiraterone acetate. The oral dose of Zytiga is 1000 mg of abiraterone acetate once daily, with 5 mg of prednisone once or twice daily [5].

The commercial product, Zytiga, has very low oral bioavailability, reportedly less than 10%. It exhibits an extremely high food effect. One study reported that a high-fat meal increased systemic exposure to abiraterone by approximately 17- and 10- fold for C_max_ and AUC_0–∞,_ respectively [7,8]. Even a low-fat meal had a substantial effect on Zytiga’s pharmacokinetics [7,8]. Since there is significant variation in diets across the patient population, the administration of Zytiga with food often leads to variable abiraterone exposure. Thus, Zytiga must be administered on an empty stomach. In addition to food effect, Zytiga also exhibits a high pharmacokinetic variability. It is reported that the inter-subject variabilities in patients with mCRPC were found to be approximately 140% for C_max_ and 107% for AUC_0–24 h_ for the single-dose Zytiga pharmacokinetics [7]. Moreover, Zytiga treatment results in subtherapeutic outcomes compared to the outcome of abiraterone if its solubility and absorption could be enhanced, such as by forming an amorphous solid dispersion. It has been reported that increased exposure to abiraterone can lead to slower disease progression, longer overall survival, and a reversal of CYP17A1 inhibition resistance [9,10,11]. However, when the dosage of Zytiga is doubled from 1000 mg to 2000 mg, only an 8% increase in the mean AUC was observed [5]. This non-linearity in dose-exposure relationship for Zytiga can largely be attributed to abiraterone acetate’s poor solubility, as evident by marked food effect. Thus, the Zytiga formulation cannot deliver enough abiraterone to achieve the maximum therapeutic effect.

Despite these reported issues associated with Zytiga, only a few attempts have been reported to enhance the solubility, dissolution, and bioavailability of abiraterone acetate. One such attempt was the development of the abiraterone acetate formulation, Yonsa^®^, that uses the SoluMatrix Fine Particle Technology™ [12,13]. Yonsa showed only a modest improvement, by doubling the bioavailability of abiraterone [14]. Other reported attempts to improve the bioavailability of abiraterone include the development of nanoamorphous formulations, lipid-based formulations, nanoparticles, and self-microemulsifying formulations [15,16,17,18,19,20]. Unfortunately, none of these attempts could yield the maximum therapeutic potential of abiraterone. Hence, there is still a need for an improved abiraterone formulation that further enhances the dissolution and pharmacokinetics of abiraterone, ultimately leading to its improved therapeutic outcome.

**Figure 2:**
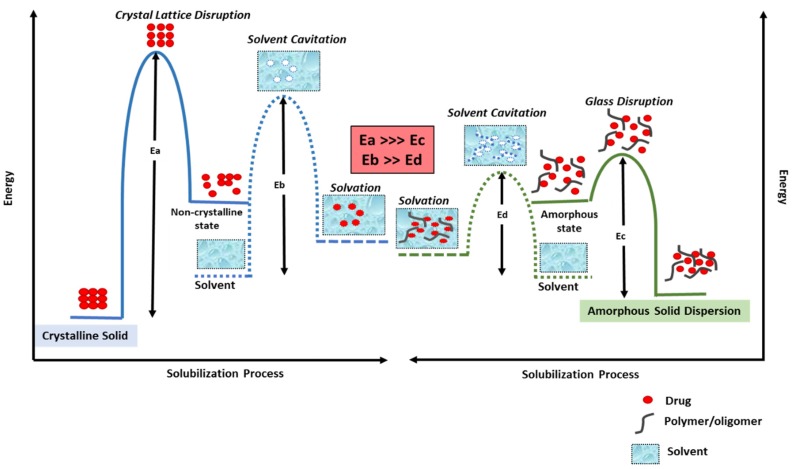
Activation energy diagram for the solubilization of a drug from a crystalline form (right) and from an ASD (left). (E_a_ is the energy required to disrupt crystalline lattice of a drug in a conventional formulation; E_c_ is the energy required to disrupt the glass solution of a drug in an ASD formulation, E_b_ is the energy required for solvent cavitation in the solubilization of a crystalline solid and E_d_ is the energy required for solvent cavitation in the case of ASD solubilization.).

An amorphous solid dispersion (ASD) results from a formulation technique in which the drug, also known as the active pharmaceutical ingredient (API), is molecularly dispersed in an inert amorphous carrier such as a polymer [21]. ASDs facilitate dissolution of poorly water-soluble drugs primarily by presenting the drug in an amorphous form, thereby lowering the total energy required for the solvation of the crystalline drug [22,23]. Figure 2 illustrates the energetics involved in solubilizing a drug, both from its crystalline form and from an ASD. The process of drug solubilization generally involves three stages: (1) disruption of the physical form of the drug (an endothermic stage), (2) solvent cavitation (also an endothermic stage), and (3) solvation of the drug (an exothermic stage) [23]. For a drug in its crystalline form, the energy required to disrupt the crystalline lattice (E_a_) is much higher than the energy required to disrupt the glass solution of a drug in an ASD form (E_c_). Also, the energy required for solvent cavitation in the solubilization of a crystalline solid (E_b_) is higher than the energy required for solvent cavitation in the case of ASD solubilization (E_d_), since the polymer/oligomer assists in higher solvent cavitation.

Thus, ASDs enhance the dissolution of poorly water-soluble drugs by lowering the energy barriers to solubilization. ASDs have been reported to enhance the pharmacokinetics of several anticancer drugs [24,25,26]. Also, ASDs have been reported to improve the therapeutic outcomes of anti-cancer drugs such as vemurafenib and gefitinib [26,27,28]. Hence, ASDs represent a promising formulation to enhance the dissolution of abiraterone and improve its pharmacokinetic properties, once a suitable manufacturing technique is found.

Several methods for manufacturing ASDs are based either on heating (e.g., hot melt extrusion (HME)) or on solvents (e.g., spray drying, conventional freeze drying, thin-film freezing, coprecipitation, electro-spinning) [29,30,31]. In the case of abiraterone, these methods are limited because of the drug’s physicochemical properties. For example, it has been reported that drugs with a high melting point ≥200 °C, like abiraterone, are difficult to render amorphous using the HME technique, thus these drugs typically lie outside the formulation space of HME [32]. Also, since HME requires the application of external heat, attempts to formulate drugs with a high melting point can lead to drug and/or polymer degradation [32,33]. Alternatively, a prerequisite for solvent-based ASD manufacturing techniques (e.g., spray drying, thin film freezing) is that the drug and polymer must be co-soluble in organic solvents or in a mixture of organic and aqueous solvents [34]. Also, solvent-based techniques pose challenges such as residual solvent toxicity and solvent explosion risks [32]. Thus, both the high melting point of abiraterone and its poor solubility in organic solvents have precluded the successful development of an abiraterone ASD, as evidenced by the absence of reports in the literature demonstrating abiraterone ASDs.

As we have previously reported, the KinetiSol^®^ technology, is a high-energy, solvent-free, thermokinetic method for manufacturing ASDs. KinetiSol does not require the application of external heat, which is a particularly useful attribute due to the general thermal sensitivity of pharmaceutical materials [35,36]. This technology can be used to develop ASDs of a wide range of drugs, including those with challenging physicochemical properties [35]. Details on KinetiSol technology are available in review manuscript by Ellenberger et al. 2018 [35]. An ideal ASD, is a system that not only achieves a high concentration of dissolved drug through the generation of a supersaturated state, but also maintains a high concentration of dissolved drug through the prevention or delay of drug precipitation. The generation of drug supersaturation is referred to as the spring effect, and its maintenance is referred to as the parachute effect [37]. Depending upon the drug’s properties, in certain cases, a binary ASD system containing the drug and a polymer (usually referred to as a primary polymer) is adequate to achieve the spring and parachute effects [38]. However, circumstances may require a ternary ASD system, which contains the drug, a primary polymer to induce the spring effect and a secondary polymer, to induce the parachute effect [39].

Thus, we hypothesize that the application of KinetiSol technology can enable the development of an ideal ASD of abiraterone and thereby improve its dissolution and pharmacokinetic properties. To investigate this hypothesis, we used long-chain polymers that are reported in development of binary KinetiSol processed amorphous solid dispersions (KSDs). In addition, we also used a short-chain oligomer for the first time. We identified the short-chain oligomer as an optimal solubility enhancer of abiraterone leading to its supersaturation (i.e., the spring effect) in acidic media. We also identified and optimized the level of a long-chain polymer to prevent abiraterone precipitation in neutral media (i.e., to create the parachute effect). We successfully developed both binary and ternary KSDs of abiraterone that improved its dissolution, increased its oral bioavailability, and reduced its pharmacokinetic variability. Therefore, the abiraterone KSD can ultimately deliver sufficient amounts of abiraterone to ensure the maximum therapeutic effect and thus improve therapeutic outcomes for prostate cancer patients.

## 2. Materials and Methods

### 2.1. Materials

Abiraterone API was purchased from Agno Pharma (New York, NY, USA). Hydroxypropyl methylcellulose of varying viscosity grades (i.e., Methocel™ E3 Premium LV, Methocel™ E5 Premium LV, Methocel™ E15 Premium LV, and Methocel™ E50 Premium LV) were purchased from the Dow Chemical Company (Midland, MI, USA). Polyvinyl pyrrolidone of varying viscosity grades (i.e., Kollidon^®^ 30 and Kollidon^®^ 90) were supplied as gift samples by BASF (Florham Park, NJ, USA). Polyvinyl acetate phthalate (i.e., Phthalavin^®^) was supplied as gift sample by Colorcon (Stoughton, WI, USA). Hydroxypropyl β cyclodextrin (i.e., Kleptose^®^ HPB) was purchased from Roquette America (Keokuk, IA, USA). Hydroxypropyl methylcellulose acetate succinate of varying degrees of acetate and succinate substitution (i.e., Affinisol™ HPMCAS 716G, Affinisol™ HPMCAS 912G, and Affinisol™ HPMCAS 126G) were supplied as gift samples by the Dow Chemical Company (Midland, Michigan, USA). Sodium carboxymethyl cellulose (i.e., Cellulose Gum 12M8P) was supplied as a gift sample by Ashland (Covington, KY, USA). Methacrylic acid and ethyl acrylate copolymer (i.e., Eudragit^®^ L 100-55) was purchased from Evonik Industries (Parisppany, NJ, USA). Microcrystalline cellulose (i.e., Avicel PH-102) was purchased from the FMC Corporation (Philadelphia, PA, USA). Mannitol (i.e., Pearlitol 200SD) was purchased from Roquette America (Keokuk, IA, USA). Cross-linked sodium carboxymethyl cellulose (i.e., Vivasol^®^) was purchased from JRS Pharma (Patterson, NY, USA). Colloidal silicon dioxide (i.e., Aerosil^®^ 200 P) was purchased from Evonik Industries (Parisppany, NJ, USA). Magnesium stearate was purchased from Peter Greven (Muenstereifel, Germany). The FaSSIF dissolution media were prepared using FaSSIF/FeSSIF/FaSSGF powder purchased from Biorelevant.com (Surrey, UK). Generic abiraterone acetate tablets (i.e., Zelgor^®^ (250mg abiraterone acetate)) were purchased from a pharmacy in India, manufactured by Sun Pharmaceutical India Ltd. (Mumbai, India). The solvents used for HPLC analysis were of HPLC grade. All other chemicals and reagents used for dissolution and HPLC analysis were of ACS grade.

### 2.2. Methods

#### 2.2.1. Development of KSDs

##### KinetiSol^®^ Processing

Abiraterone ASDs were prepared using a KinetiSol small-scale compounder (formulator) designed and manufactured by DisperSol Technologies LLC (Georgetown, TX, USA). Before compounding, the API and polymer/oligomer excipients were accurately weighed, dispensed into a polyethylene bag, and hand-blended for 2 min to prepare physical mixtures (PMs). These physical mixtures were charged into the KinetiSol formulator chamber. Inside the formulator chamber, a shaft with protruding blades was rotated at varying incremental speeds ranging from 4000 rpm to 6000 rpm without the addition of external heat in order to impart frictional and shear forces to the sample material. The temperature of the mass was monitored using an infrared probe. When the molten mass temperature reached 160 °C, the mass was rapidly ejected, collected, and pressed between two stainless steel plates to rapidly quench the sample.

##### Milling

The quenched mass obtained after KinetiSol processing was milled using a lab scale rotor mill (i.e., IKA tube mill 100 (IKA Works GmbH & Co. KG, Staufen, Germany)). For milling, the fragments of quenched mass were loaded into a 20 mL grinding chamber, which was operated for 60 s with a grinding speed between 10,000 and 20,000 rpm. This milled material was subsequently passed through a #60 mesh screen (≤250 μm). Material retained above the screen (i.e., >250 μm) was cycled through the mill with the same parameters. This process of milling and sieving was repeated until all material passed through the screen. The resultant material (<250 μm) was labeled as KSD.

#### 2.2.2. Physicochemical Characterization of KSDs

##### X-Ray Powder Diffraction

X-Ray powder diffraction (XRPD) analysis was conducted using a Rigaku MiniFlex600 II (Rigaku Americas Corporation, The Woodlands, TX, USA) instrument equipped with a Cu-Kα radiation source generated at 40 kV and 15 mA. The API, PM, and KSD samples were loaded into an aluminum pan, leveled with a glass slide, then analyzed in the 2-theta range between 2.5° and 35.0° while being spun. The step size was 0.02°, and the scanning rate was set to 5.0°/min. The following additional instrument settings were used: Slit condition: variable + fixed slit system; soller (incident): 5.0 degrees; IHS: 10.0 mm; DS: 0.625 degrees; SS: 8.0 mm; soller (receiving): 5.0 degrees; RS: 13.0 mm (open); and monochromatization: kb filter (×2). The data were collected using Miniflex Guidance software (Rigaku Corporation, Tokyo, Japan) and processed using PDXL2 software (Rigaku Corporation, Tokyo, Japan).

##### Modulated Differential Scanning Calorimetry

Thermal analysis was conducted with modulated differential scanning calorimetry (mDSC) using a differential scanning calorimeter model Q20 (TA Instruments, New Castle, DE, USA) equipped with a refrigeration-based cooling system and an autosampler. The API and KSD samples were prepared by weighing 5–10 mg of the material and loading it into a Tzero pan. The pan was sealed with a Tzero lid using a Tzero press. Following the sample equilibration at 30 °C for 5 min, the temperature was ramped at 5 °C/min up to 250 °C with a modulation of ±1 °C every 60 s. Nitrogen was used as the sample purge gas at a flow rate of 50 mL/min. The data were collected using TA Instruments Explorer software (TA Instruments, New Castle, DE, USA) and processed using Universal Analysis software (TA Instruments, New Castle, DE, USA).

##### HPLC Analysis

High-performance liquid chromatography (HPLC) methods were developed for the chemical analysis of abiraterone KSDs. An Agilent HPLC system-1260 Infinity (Agilent, Santa Clara, CA, USA) was used for reverse phase HPLC analysis. The HPLC column was a Zorbax C18 extend (150 mm × 4.6 mm, 3.5 µm) (Agilent, Santa Clara, CA, USA).

For binary KSDs, mobile phase A was 0.1% formic acid, and mobile phase B was degassed acetonitrile. A gradient profile was designed. Initially, mobile phase A was set at 70%, at 15 min mobile phase A was set at 50%, at 20 min mobile phase A was set at 40%, at 25 min mobile phase A was set at 30%, at 30 min mobile phase A was set at 10%, at 35 min mobile phase A was set at 50% and at 40 min mobile phase A was set back to 70%. The flow rate was 0.5 mL/min and the run time was 40 min. The column was held at 25 °C, and the data were collected at a single wavelength of 254 nm. The standard drug solutions were prepared in concentration range of 500 µg/mL to 0.98 µg/mL. The calibration curve with R^2^ value of 0.99 was obtained. The abiraterone retention time was ~20.4 min. Samples were prepared at a nominal concentration of 100 µg/mL level with 7:2:1 acetonitrile:methanol:formic acid (0.1%) as the standard or sample diluent. All samples were filtered through 0.45 μm nylon syringe filters (GE Healthcare Bio-Sciences, Piscataway, NJ, USA), before analysis. Sample chromatography was analyzed using Empower software, version 3.0 (Waters, Milford, MA, USA).

For ternary KSDs, mobile phase A was 0.005M Diammonium phosphate solution, and mobile phase B was degassed methanol. An isocratic profile with 25:75 Mobile phase A: Mobile phase B was designed. The flow rate was 1.5 mL/min and the run time was 20 min. The column was held at 40 °C, and the data were collected at a single wavelength of 254 nm. The standard drug solutions were prepared in concentration range of 1000 µg/mL to 1.95 µg/mL. The calibration curve with R^2^ value of 1.0 was obtained. The abiraterone retention time was ~9.8 min. Samples were prepared at a nominal concentration of 100 µg/mL level with methanol as the standard or sample diluent. All samples were filtered through 0.45 μm nylon syringe filters (GE Healthcare Bio-Sciences, Piscataway, PE, USA), before analysis. Sample chromatography was analyzed using Empower software, version 3.0 (Waters, Milford, MA, USA).

##### Dissolution

An in vitro, non-sink, gastric transfer dissolution method was developed to analyze the dissolution of abiraterone API, the generic abiraterone acetate tablets, the binary KSDs, to select ternary component for KSDs, as well as to analyze the dissolution of the ternary KSDs. For the dissolution analysis of abiraterone API and the binary and ternary KSDs, the samples (equivalent to 31 mg of abiraterone API) were loaded in an Erlenmeyer flask (dissolution vessel) containing 35 mL of 0.01N HCl (pH 2.0), placed in an incubator–shaker (Excella E24 (New Brunswick Scientific, Edison, NJ, USA)) set to 37 °C and a rotational speed of 180 rpm. After 30 min, 35 mL of FaSSIF (prepared in a 50 mM phosphate buffer at pH 6.8) was added to the dissolution vessel. At predetermined time points, samples were drawn from the dissolution vessel and centrifuged using an ultracentrifuge at 92,000 rpm for 90 s (Airfuge™, Beckman, Indianapolis, IN, USA). The supernatants were further diluted using the HPLC diluent and analyzed using the respective HPLC methods mentioned above. For the generic abiraterone acetate tablets, the whole tablet was analyzed, and the dissolution media volumes were scaled accordingly. For the selection of the ternary component, 35 mg of secondary polymer candidates were added in 35 mL of 0.01 N HCl (pH 2.0), and the dissolution of the binary KSD was conducted using the method described above. The area under the drug dissolution curve (AUDC) was calculated using the linear trapezoidal method.

##### Tableting

The KSD and tableting excipients (Avicel PH-102, Pearlitol 200SD, Vivasol, Aerosil 200 P and magnesium stearate) were accurately weighed and dispensed. Aerosil 200 P was sieved through #40 mesh (420 µm) until all material passed through the sieve. The KSD and all tableting excipients, except magnesium stearate, were loaded in a vial and mixed using a vortex mixer (Thermo Scientific, Waltham, MA, USA). Magnesium stearate was then added to the vial and blended using a spatula. The resultant tableting blend was then dispensed in aliquots equivalent to 44.6 mg of abiraterone. Each aliquot was loaded in the tablet die having dimensions of 0.3740 × 0.7480 inches and a modified oval shape. The tablets were compressed using a single-station hand tablet press (BVA Hydraulics, Kansas City, MO, USA) with a compression pressure of 800–900 psi, dwell time of <2 s and target hardness of 8–12 kP.

##### Pharmacokinetic Study in Beagle Dogs

An in vivo pharmacokinetic study in fasted non-naïve male beagle dogs was carried out at Charles River Laboratories (Wilmington, MA, USA). This animal study was conducted according to an approved Charles River Laboratories IACUC protocol (#20111395, September 2017). The 44.6 mg equivalent abiraterone tablets were analyzed along with a generic 250 mg equivalent abiraterone acetate tablet. Each study arm for each formulation consisted of five dogs. The dogs were fasted overnight before dosing, and the food was returned after 4 h post dosing. Each dog was administered a single tablet of the respective formulation (as per the study arm) along with a post dose flush of 40 mL sterile water adjusted to pH 2.0. At predefined time points of 0.5, 1, 1.5, 2, 3, 4, 6, 8, and 10 h post dose, 1 mL blood samples were drawn from each dog using venipuncture of a peripheral vessel and placed into tubes containing sodium heparin anticoagulant. The blood samples were centrifuged to isolate the plasma. The plasma samples were then analyzed using liquid chromatography with tandem mass spectrometry (LC-MS/MS) for abiraterone content.

For LC-MS method, the samples were analyzed on Shimadzu LC-30AD system (Shimadzu, Kyoto, Japan). The analytical column used was Phenomenex Luna Omega Polar C18 1.6 µm 2.1 × 50 mm (Phenomenex, Torrance, CA, USA). The mobile phase A was 10 mM ammonium acetate and mobile phase B was 2% IPA in acetonitrile. A 1.00 min gradient was utilized going from 2% to 100% of Mobile Phase B for a total run time of 4.00 min. The flow rate was 0.6 mL/min and the injection volume was 1 µL. The primary stock solution was prepared at 1000 µg/mL in 100% methanol. Calibration standard spiking solutions were prepared at 25.0, 50.0, 100, 200, 400, 800, 1600, 3200, 6400, 12,800, 25,600, 51,200, and 102,000 ng/mL in 100% DMSO. Matrix calibration standards were prepared at 0.250, 0.500, 1.00, 2.00, 4.00, 8.00, 16.0, 32.0, 64.0, 128, 256, 512, and 1020 ng/mL in plasma sodium heparin. For Mass spectroscopy AB Sciex Qtrap 6500 system (Sciex, Redwood City, CA, USA), with Multiple Reaction Monitoring (MRM) scan and unit resolution was used. The mass spectrometer was equipped with electrospray ionization interface, operated in positive ion mode, the dwell time was 55 ms and the m/z was 350.194/334.176 Da.

##### Pharmacokinetic Analysis

Pharmacokinetic parameters were estimated using Watson pharmacokinetic software version 7.3.0.01 (Thermo Fisher Scientific, Waltham, MA, USA) using a non-compartmental approach consistent with the oral route of administration. The area under the plasma concentration–time curve (AUC) was calculated using the linear trapezoidal method. The relative bioavailability (i.e., the F value) was calculated using the following formula:

$$F=\frac{{\mathrm{AUC}}_{\left(0-10\mathrm{hr}\right)\left(\mathrm{test}\;\mathrm{abiraterone}\;\mathrm{tablet}\right)}\times {\mathrm{Dose}}_{\left(\mathrm{abiraterone}\;\right)\left(\mathrm{generic}\;\mathrm{abiraterone}\;\mathrm{acetate}\;\mathrm{tablet}\right)\;}}{{\mathrm{AUC}}_{\left(0-10\mathrm{hr}\right)\left(\;\mathrm{generic}\;\mathrm{abiraterone}\;\mathrm{acetate}\;\mathrm{tablet}\right)}\times {\mathrm{Dose}}_{\left(\mathrm{abiraterone}\;\right)\;\left(\mathrm{test}\;\mathrm{abiraterone}\;\mathrm{tablet}\right)}}$$

Statistical analysis was performed using JMP^®^ 14.3.0 software (SAS, North Carolina, USA). The two groups were compared consecutively using Student’s *t*-test (α = 0.05).

## 3. Results and Discussion

### 3.1. Development of Binary KSDs

In order to develop binary KSDs of abiraterone, we selected five polymers/oligomers that vary in their chemistry, architecture, molecular weight, and viscosity (see Table 1). We selected the primary polymers/oligomers with varying chemistry and architecture (long-chain linear and short-chain cyclic architectures) to enable all possible noncovalent interactions between the primary polymer/oligomer and abiraterone in the binary KSDs. This was done to determine the most suitable primary polymer/oligomer that would form a stable, molecularly dispersed abiraterone KSD and prevent abiraterone recrystallization. We selected primary polymers/oligomers that have a wide range of molecular weights and viscosity grades to explore the thermokinetic processing space of KinetiSol technology. Table 2 lists the binary KSDs’ composition, processing parameters, and appearance after KinetiSol processing.

All primary polymer compositions (i.e., the Lots 1, 2, 3, and 4 PMs) were found to be processable using KinetiSol technology.

From the literature it can be observed that so far only polymers have been used for KinetiSol processing [35]. One of the reasons for this is that KinetiSol technology emerged from the plastics industry, where process feeds consist chiefly of plastics/polymers [36]. The most likely reason for the focus on polymers for KinetiSol processing is based on generation of frictional and shear stresses necessary for thermal fusion of PM components during the process itself [36]. During the induction stage of KinetiSol processing, solid particles collide and generate heat through friction. It can be assumed that short-chain, low molecular weight oligomers offer lower frictional resistance as compared to long-chain, high molecular weight polymers. Also, thermal conductivity of long-chain linear polymers is higher than short-chain cyclic oligomers. This is because the conformational energy due to covalent bonds (dominated in polymers) is critical for thermal conductivity than non-bonding energy due to van-der-Waal interactions (dominated in oligomers) [40]. Following the induction phase, mixing occurs, and the semi-molten components form a mass of material, which is compounded by the KinetiSol mixing elements. At this stage, the shear forces dominate the process and are critical for molecularly dispersing the API in the carrier excipient. The power law for non-Newtonian fluids can be applied to the semi-molten components, wherein the shear stress is directly proportional to fluid consistency [41]. Since, the consistency of the oligomer melt is lower than that of a polymer melt, it can be assumed that processing of oligomer does not generate sufficient shear energy. However, in the present study, it was demonstrated for the first time that Lot 5 PM containing HPBCD, which is a cyclic oligomer with low molecular weight, was also well processed using KinetiSol technology. The probable reason for this is that the thermal mobility of oligomers is higher than that of polymers, as a result, the surface contact area for oligomers is higher, yielding sufficient friction during induction phase. Another reason is that, high affinity between drug and oligomer aids in building up sufficient fluid consistency, which generates necessary shear stress leading to optimal KinetiSol processing.

The total processing time for all the lots was less than 45 s. The processing time at elevated temperatures for the Lots 1–4 KSDs was less than 15 s, while the Lot 5 KSD required less than 7.5 s. Discoloration was observed in the Lots 1–4 KSDs but not in the Lot 5 KSD. This indicates that the discoloration was most likely due to long-chain polymer processing at elevated temperatures. The Lot 1 and Lot 2 KSDs showed slight discoloration and were yellowish in color, while the Lot 3 KSD also showed slight discoloration but was light brown in color. None of these KSDs showed high levels of discoloration consistent with polymer degradation, as was seen during HME of these polymers [42,43]. The Lot 4 KSD showed significant discoloration and was brown in color. This could be due to PVAP degradation at elevated temperatures and also due to subsequent drug degradation due to interaction with polymer degradants [44]. All the lots showed an opaque appearance, as expected, due to trapped air in the ejected KSD masses. The binary KSD masses for the Lots 1–4 KSDs were more agglomerated and less brittle than the mass of the Lot 5 KSD, since long-chain linear polymers were used in the Lots 1–4 KSDs, which imparted a more rigid network of polymers within which abiraterone was dispersed. Nonetheless, all the binary KSD masses were successfully milled to yield a binary KSD powder with a particle size of <250 µm.

### 3.2. Physicochemical Characterization of Binary KSDs

**Figure 3:**
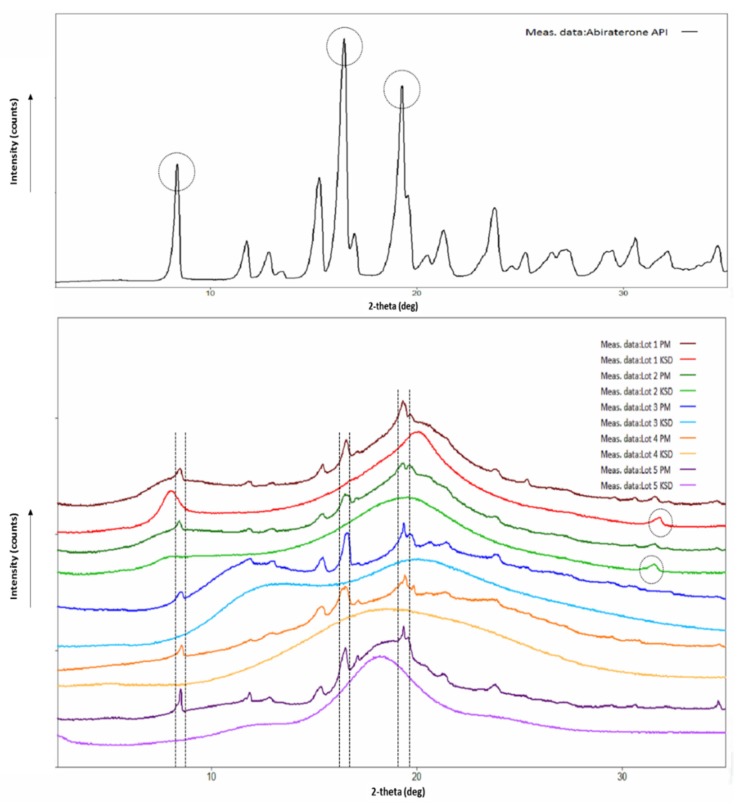
X-Ray diffractograms of abiraterone API (top) and abiraterone binary PMs, KSDs (bottom).

Burke et al. reported that abiraterone exists in the crystalline state, and its crystal conformation is stabilized by hydrogen bonding between the nitrogen atom of the pyridine ring and the hydroxyl group [48]. The X-ray diffractogram of abiraterone showed sharp diffraction peaks (see Figure 3), indicating its crystalline state. Three main diffraction peaks for abiraterone were observed at 8.38°, 16.46° and 19.27° that were similar to those reported by Solymosi et al. [6]. However, certain peak positions and relative peak intensities of our abiraterone API X-ray diffractogram differed from that reported by Solymosi et al. [6]. This could be due to differences between sample packing and/or the polymorphs of abiraterone [16]. These diffraction peaks of abiraterone were also observed in the X-ray diffractogram (XRD) of binary PMs (see Figure 3), thus indicating their crystalline nature. The X-ray diffractograms of all the binary KSDs showed a halo pattern and no abiraterone diffraction peaks (see Figure 3), thus indicating their amorphous nature. In the diffractograms of the Lots 1 and 2 KSDs, a very small diffraction peak was observed around 31.5–31.7°. This peak was also observed in the diffractograms of the Lots 1 PM, 2 PM, HPMC E3, and HPMC E5 (data not shown). We noted that both HPMC E3 and HPMC E5 contain ≤1% sodium chloride [49]. Lee et al. observed a sharp diffraction peak at 31.5° in the X-ray diffractogram of sodium chloride [50]. Therefore, the diffraction peak seen around 31.5–31.7° in the diffractograms of the Lots 1 and 2 KSDs is attributed to the presence of sodium chloride.

**Figure 4:**
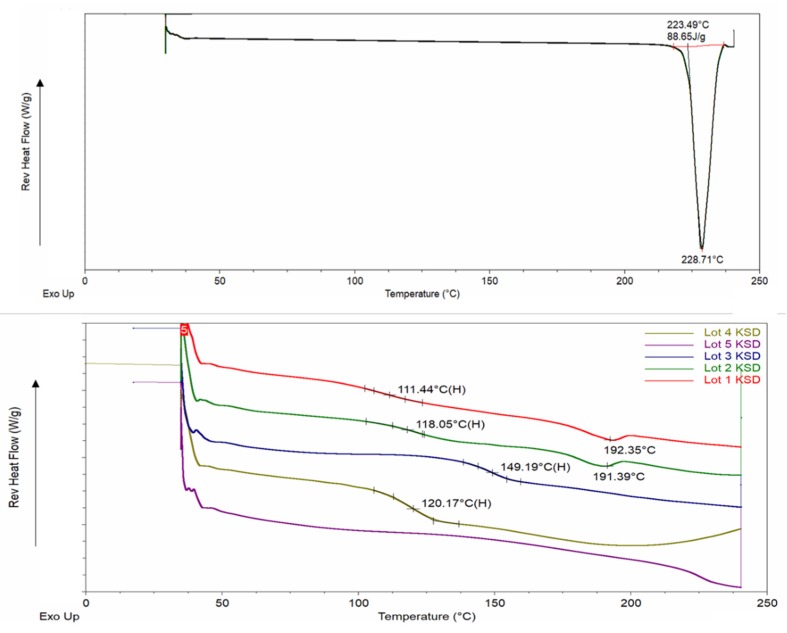
mDSC thermograms of abiraterone API (top) and abiraterone binary KSDs (bottom).

The mDSC thermogram of abiraterone (see Figure 4), showed a sharp melting endotherm at 228.71 °C. This confirmed a melting point and melting range of 501 K (227.85 °C) and 228–230 °C, respectively, which has been reported in the literature [6,51]. None of the mDSC thermograms of binary KSDs showed any melting endotherms (see Figure 4). In particular, there was no melting endotherm at 228 °C. This further substantiates that KinetiSol processing rendered abiraterone amorphous in all the binary KSDs. The Lots 1–4 KSDs showed a glass transition temperature (T_g_) of ~111, ~118, ~149, and ~120 °C, respectively. No other T_g_ event was observed for these lots. Thus, we can infer that these KSDs are largely single-phase systems in which abiraterone is uniformly and homogeneously dispersed in the respective polymers. For the Lot 1 and Lot 2 KSDs, a small thermal event was observed at 192.35 °C and 191.39 °C, respectively. The magnitude of these thermal events was negligible, and they may be an artifact of the experimental parameters or may be indicative of trace crystallinity. For the Lot 5 KSD, no thermal event was observed, which indicates that a single-phase amorphous system was formed.

HPLC analysis showed that the KSDs of Lots 1, 2, 3, and 5 had purities of 99.2, 99.2, 97.5, and 99.3%, respectively. The KSDs of Lots 1, 2, and 5 showed no individual impurity ≥0.5%. The Lot 3 KSD showed two unknown impurities of >0.5% but <1.0%. These data demonstrate that no significant degradation of abiraterone API occurs during KinetiSol processing. The Lot 4 KSD showed total impurities of >5.0%. This could be due to PVAP having undergone significant degradation, which led to the formation of phthalic acid and acetic acid, thus causing abiraterone degradation [44,52]. A polymer similar to PVAP (i.e., cellulose acetate phthalate) undergoes thermal degradation at 105 °C to form phthalic acid and acetic acid [53].

### 3.3. Dissolution of Binary KSDs

**Figure 5:**
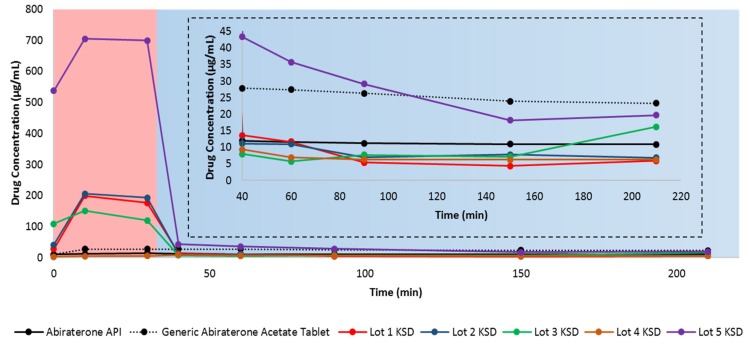
In vitro, non-sink, gastric transfer dissolution profiles of neat abiraterone API, generic abiraterone acetate tablets, and binary KSDs. The red region: 0.01 N HCl. The blue region: FaSSIF. The inset represents the enlargement of the dissolution profile in FaSSIF.

For dissolution testing, we employed a two-stage, non-sink, gastric transfer dissolution method in which the sample is first exposed to acidic media, simulating fasted stomach content, followed by exposure to neutral-to-basic media, simulating fasted intestinal content. Figure 5 illustrates the in vitro, non-sink, gastric transfer dissolution profile of abiraterone API, the generic abiraterone acetate tablet, and the binary KSDs.

With the exception of the Lot 4 KSD, all other binary KSDs were able to enhance the overall dissolution of abiraterone compared to the neat crystalline abiraterone API. After integrating the total area under the drug dissolution curve (AUDC_Total_), we found that the relative AUDC_Total_ for the generic abiraterone acetate tablet was 219.8% relative to neat abiraterone API. This difference occurs because abiraterone acetate is more soluble than abiraterone in biorelevant media [2,6].

The relative AUDC_Total_ for the Lot 1 KSD, the Lot 2 KSD, and the Lot 3 KSD was 288.8%, 316.0%, and 255.1%, respectively, relative to neat abiraterone API. This shows that the KSDs based on the polymers HPMC E3, HPMC E5, and PVP K30 can enhance abiraterone dissolution even more than its prodrug abiraterone acetate due to abiraterone amorphization. The relative AUDC_Total_ for the Lot 4 KSD was just 55.1% as compared to neat abiraterone API, indicating that PVAP-based KSDs showed lower abiraterone dissolution than even the crystalline abiraterone API. However, this is due to the higher impurities in Lot 4 KSD. As discussed above, the PVAP was degraded in this lot, leading to a potential loss of ionizable groups, which in turn lowers both PVAP solubilization and abiraterone solubilization. The relative AUDC_Total_ for the Lot 5 KSD was dramatically higher (i.e., 1173.4%) relative to abiraterone (as the neat API). This indicates that the oligomer HPBCD-based KSD far surpassed the dissolution enhancement of neat abiraterone API when compared not only to its prodrug abiraterone acetate but also to its polymer-based KSDs.

HPBCD has a hydrophobic inner cavity and a hydrophilic outer surface. It can form an inclusion complex with poorly water-soluble drugs by interacting with the hydrophobic groups of the drug and including them within its cavity. This interaction improves the drug’s physicochemical properties and thus increases the drug’s aqueous solubility [54]. Oligomer-based KSD dissolution performance surpassed that of polymer-based KSDs, likely because in addition to abiraterone amorphization in the Lot 5 KSD, HPBCD further enhanced abiraterone solubility by forming inclusion and non-inclusion complexes with abiraterone, likely in KSD and during dissolution [54,55]. Moreover, the oligomer HPBCD has a more hydrophilic outer surface than the other polymers studied, thereby leading to higher abiraterone hydration and solubilization. Similarly, Verma and Kumar reported higher gliclazide dissolution in the HPBCD solid dispersion than the PVP K30 solid dispersion [56].

Abiraterone was more soluble in acidic media (i.e., in 0.01 N HCl) and less soluble in neutral to basic media (i.e., when FaSSIF was added). This is because abiraterone is a weakly basic drug. Solymosi et al. also observed that abiraterone was more soluble in simulated gastric fluid (pH 1.6) than FaSSIF [6]. The generic abiraterone acetate tablet is more soluble than abiraterone API in acidic media. Upon closer examination, it is evident that Lots 1, 2, 3, and 5 abiraterone KSDs have higher dissolution, meaning that they can attain more supersaturation of abiraterone compared to neat abiraterone API and the generic abiraterone acetate tablet in 0.01 N HCl.

However, rapid abiraterone precipitation occurs for KSDs in FaSSIF media. This means that HPMC E3, HPMC E5, PVP K30, and HPBCD were acceptable spring agents in 0.01 N HCl but were poor parachute agents in FaSSIF media. The majority of the abiraterone will precipitate as crystalline abiraterone due to its higher crystallization tendency. However, some of the abiraterone can precipitate into an amorphous aggregate form. For example, during the dissolution of enzalutamide ASDs, the formation of drug crystals and amorphous drug aggregates have been observed [57]. The mechanism of abiraterone precipitation in these conditions requires further investigation. The degree of precipitation from 0.01 N HCl to FaSSIF for all the KSDs discussed above has a similar range, from 92.0–94.0%. This suggests that neither HPMC E3, HPMC E5, PVP K30, nor HPBCD are good precipitation inhibitors. This may be due to (a) the low viscosity grades of HPMC E3, HPMC E5, and PVP K30 and (b) the low molecular weight and cyclic architecture of HPBCD. The drug concentration of the generic abiraterone acetate tablets in FaSSIF media was 25.74 µg/mL on average, which is similar to the abiraterone acetate FaSSIF solubility value of 64.6 ± 5.2 µM (i.e., ~25.29 µg/mL) reported in the literature [2]. Interestingly, in another study, the FaSSIF solubility of abiraterone acetate from Zytiga was reported to be only 18 µg/mL [15]. We observed that the average solubility of abiraterone API in FaSSIF was 11.33 µg/mL, which conflicts with the value of 12.7 ± 4.2 µM (i.e., ~4.43 µg/mL) reported previously [2]. This is likely due to differences in abiraterone polymorphs, as discussed above. Of all KSDs, in FaSSIF media, only the Lot 5 KSD had a higher drug concentration than abiraterone API. But, when compared to the drug concentration of generic abiraterone acetate tablets in FaSSIF media, the Lot 5 KSD exhibited higher drug concentration only up to 90 min.

Thus, it was determined that of all the binary KSDs tested, the Lot 5 KSD containing HPBCD showed the highest overall dissolution enhancement for abiraterone. HPBCD showed a significant spring effect in 0.01 N HCl but showed a poor parachute effect in FaSSIF media. Hence, the precipitation of abiraterone in FaSSIF media must be inhibited or reduced.

### 3.4. Selection of a Suitable Ternary Component for KSDs

The main goal of adding a ternary component (i.e., a secondary polymer) to the KSDs in the current study is to prevent abiraterone precipitation in FaSSIF media. For this reason, we decided to explore two options.

First, we selected secondary polymer candidates that dissolve immediately and impart high viscosity in the microenvironment of the dissolving drug at lower polymer concentrations. The rationale here is that polymers that have a higher viscosity grade would maintain supersaturated drug concentrations and prevent the drug’s precipitation in FaSSIF.

It has been reported that an increase in the viscosity of the media reduces molecular mobility, thereby interfering with drug nucleation and crystallization [58,59]. Moreover, higher-viscosity grade polymers tend to have higher molecular weights and more functional groups that interact with the precipitated hydrophobic crystalline drug surface, thus preventing further drug crystallization [58].

Hence, we selected the following: (a) hydroxypropyl methylcellulose (Methocel™ E15 Premium LV (HPMC E15)), (b) Methocel™ E50 Premium LV (HPMC E50), (c) polyvinyl pyrrolidone (Kollidon^®^ 90 PVP K90), and (d) sodium carboxymethyl cellulose (Cellulose Gum 12M8P (Na CMC)). A 2% *w/v* aqueous solution of HPMC E15 and HPMC E50 imparts a viscosity of 15 mPa·s and 50 mPa·s, respectively [45]. A 10% *w/v* aqueous solution of PVP K 90 can impart a viscosity of 300–700 mPa·s [45]. A 1% *w/v* aqueous solution of Na CMC can impart a viscosity of about 2000 mPa·s [45].

Secondly, we selected polymers that have pH-dependent solubility and are soluble at pH 5 and above. The rationale here is that these polymers dissolve in FaSSIF, then increase viscosity in the microenvironment of the dissolving drug, and thus prevent drug precipitation. Therefore, we selected hydroxypropyl methylcellulose acetate succinate with varying degrees of acetate and succinate substitution (i.e., Affinisol™ HPMCAS 716G, Affinisol™ HPMCAS 912 G, and Affinisol™ HPMCAS 126 G), Polyvinyl acetate phthalate (i.e., Phthalavin^®^ (PVAP)) and methacrylic acid-ethyl acrylate copolymer (i.e., Eudragit^®^ L 100-55). HPMCAS 716 G, HPMCAS 912 G, and HPMCAS 126 G dissolve at a pH of ≥5.5, ≥6.0, and ≥6.8, respectively [35,60]. PVAP dissolves at a pH of ≥5.0, and Eudragit^®^ L 100-55 dissolves at pH of ≥5.5 [45].

**Figure 6:**
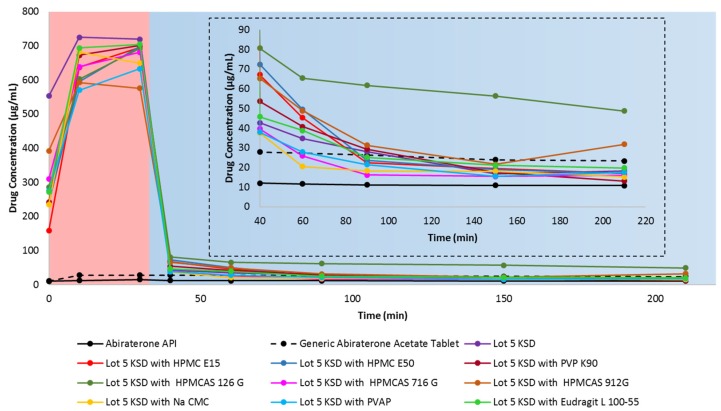
In vitro, non-sink, gastric transfer dissolution profile of abiraterone API, generic abiraterone acetate tablets, Lot 5 KSD and Lot 5 KSD with different secondary polymer candidates. Red region: 0.01 N HCl. Blue region: FaSSIF. The inset represents the enlargement of the dissolution profile in FaSSIF.

Figure 6 shows the in vitro, non-sink, gastric transfer dissolution profiles of the Lot 5 KSD with different secondary polymers. Table 3 lists the values for the relative area under the drug dissolution curve. It is important that secondary polymers prevent abiraterone precipitation in FaSSIF media, but it is also important that they do not significantly decrease abiraterone supersaturation in 0.01 N HCl.

It has been reported that the addition of a hydrophilic polymer typically increases a cyclodextrin’s solubilization ability [61]. However, from the AUDC_0.01 N HCl_ values relative to the Lot 5 KSD, it is evident that the addition of secondary polymer candidates reduced abiraterone dissolution by ≤22.0%. This could result from the hydrophilic groups of these polymers interacting with the outer surface of HPBCD, thereby reducing its ability to enhance dissolution. Interestingly, even for polymers dissolving at pH ≥ 5, there was a reduction in AUDC_0.01 N HCl_ values relative to the Lot 5 KSD. The cause of this needs further investigation.

According to Figure 6, most secondary polymer candidates prevent abiraterone precipitation in FaSSIF media. However, HPMCAS 126 G was the most capable of preventing abiraterone precipitation. The AUDC_FaSSIF_ value of HPMCAS 126G was 244.8% relative to Lot 5 KSD, thereby making HPMCAS 126G the best precipitation inhibitor (or the best parachute agent) among the secondary polymer candidates that were tested. This could result from HPMCAS 126 G dissolving above pH ≥ 6.8, thereby dissolving only in FaSSIF media to exert its effect. It is reported that HPMCAS is amphiphilic [62]. Its hydrophobic regions interact with abiraterone, and its hydrophilic regions interact with FaSSIF media, and permit the stabilization of abiraterone. Also, among the various grades of HPMCAS tested, HPMCAS 126 G was the most hydrophobic. Its relatively higher substitution with hydrophobic methoxy and acetate groups can interact with the hydrophobic regions of abiraterone and prevent further recrystallization [63]. It has been reported that HPMCAS maintains drug supersaturation and prevents drug precipitation by reducing molecular mobility for nucleation, thus prolonging the time required for nucleation and re-dissolving precipitated aggregates by interacting with the hydrophobic groups on the surface of the drug, hence interfering with drug crystallization [64,65,66]. So, it was determined that HPMCAS 126 G was a suitable secondary polymer for the development of a ternary abiraterone KSD.

### 3.5. Development of Ternary KSDs

We developed several compositions of ternary KSDs to identify the suitable concentration of HPBCD and HPMCAS 126 G to achieve the highest dissolution of abiraterone. Table 4 provides the composition of the ternary KSDs, along with their processing parameters and appearance. We observed that ternary KSD compositions (i.e., the Lots 6, 7, and 8 PMs) could be processed using KinetiSol technology. The Lot 9 PM appeared to be under-processed in the defined conditions, likely because Lot 9 PM contained more HPMCAS 126G, leading to more friction and faster attainment of the set temperature of 160 °C. Thus, the Lot 9 KSD had the lowest processing time, which was not sufficient for fusion of the KSD components. However, this assumption is based only on appearance and was verified by XRPD analysis.

Overall, the shear stress stages and processing times for all ternary KSDs were less than the binary KSD (i.e., the Lot 5 KSD) (see Table 2). The total processing time of the Lots 6 to 9 KSDs was less than 20 s, and the processing time at elevated temperature was less than 5 s. The Lots 6, 7, 8, and 9 KSDs showed slight discoloration and were light brown in color. As expected, these lots were opaque due to air entrapment in the KSD masses. All the ternary KSD masses were successfully milled to yield ternary KSD powders with a particle size of <250 µm.

### 3.6. Physicochemical Characterization of Ternary KSDs

**Figure 7:**
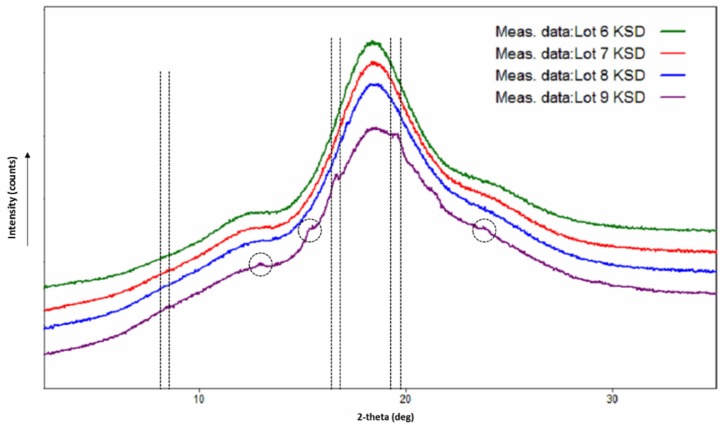
X-ray diffractograms of abiraterone ternary KSDs.

The X-ray diffractograms of the Lots 6, 7, and 8 KSDs (see Figure 7) showed a halo pattern and no abiraterone diffraction peaks, indicating their amorphous nature. The X-ray diffractogram of the Lot 9 KSD showed characteristic abiraterone peaks at 16.65° and 19.55°, but did not show the peak at 8.38°, which suggests a crystalline nature or a partially amorphous nature. The X-ray diffractogram of the Lot 9 KSD also showed small peaks at 13.08, 15.47, and 23.82°, which are also present in the crystalline abiraterone API diffractogram (Figure 3). Thus, as noted above, (based on appearance) the Lot 9 KSD was under processed.

We analyzed only the Lots 6, 7, and 8 KSDs for purity because they were amorphous. The HPLC analysis showed that the Lots 6, 7, and 8 KSDs had purities of 98.1%, 98.2%, and 98.5%, respectively. The Lots 6, 7, and 8 KSDs showed no individual impurity ≥0.5%. The total impurity level of these lots was only slightly higher than the Lot 5 KSD. This could occur because at higher temperatures, HPMCAS 126G can degrade to form acetic acid and succinic acid [60]. In the case of KinetiSol processing, the amount of HPMCAS 126G degradation would have been minimal due to only transient exposure to high temperatures. Thus, low levels of acid would be released, leading to a negligible level of abiraterone impurity formation.

### 3.7. Dissolution of Ternary KSDs

**Figure 8:**
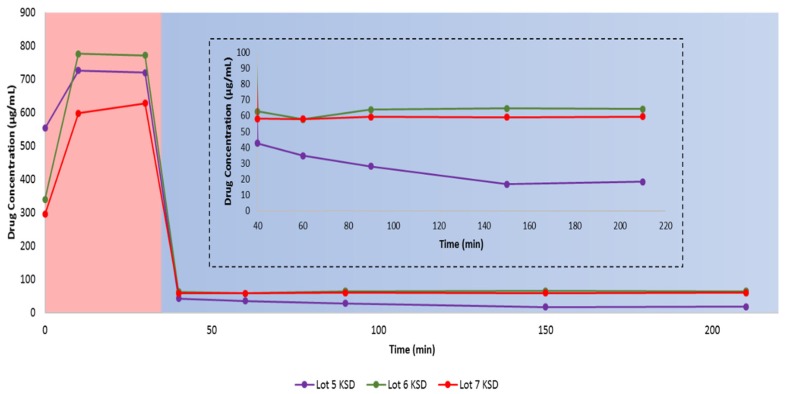
In vitro, non-sink, gastric transfer dissolution profiles of the Lots 5, 6, and 7 KSDs. Red region: 0.01 N HCl. Blue region: FaSSIF. The inset represents the enlargement of the dissolution profile in FaSSIF.

Initially, we conducted dissolution of the ternary KSDs (i.e., the Lots 6 and 7 KSDs) and compared this to the binary KSDs (i.e., the Lot 5 KSD) (see Figure 8). The overall dissolution of the Lot 6 KSD > Lot 5 KSD > Lot 7 KSD. The initial supersaturation of abiraterone in 0.01 N HCl was higher for the Lot 6 KSD > Lot 5 KSD > Lot 7 KSD. The reason for this can be attributed to the mole ratio of abiraterone:HPBCD present in the KSDs. The mole ratio of abiraterone:HPBCD in the Lots 5 and 6 KSDs was 1.00:2.25 and 1.00:2.00, respectively, while the mole ratio for the Lot 7 KSD was 1.00:1.75. Thus, in the Lot 7 KSD, there were insufficient moles of HPBCD to interact with or include the abiraterone molecule, leading to lower supersaturation levels. The exact nature of the interaction between abiraterone and HPBCD in KSDs needs further investigation. Contrary to the dissolution behavior observed in 0.01 N HCl during the selection of the ternary component (see Figure 6), the addition of HPMCAS 126 G within the ternary KSD led to better supersaturation in 0.01 N HCl, as was expected [61]. This occurs because HPMCAS 126 G was added externally to the KSD during the process of selecting the ternary component, but in the ternary KSDs, HPMCAS 126 G was included in the KSD, where it could interact with abiraterone and aid in dissolution. From the dissolution in FaSSIF, it is evident that increasing the concentration of HPMCAS 126 G did not further increase precipitation inhibition or the parachute effect. Thus, it was determined that the ternary KSD composition of 10:80:10 (% *w/w*) of abiraterone:HPBCD:HPMCAS 126 G was the most suitable formulation to enhance abiraterone dissolution.

Sarode et al., reported that the thermal processing (HME) of HPMCAS-H grade (similar to HPMCAS 126 G) had a negative impact on drug dissolution due to the degradation of HPMCAS-H and the release of free acids. Since KinetiSol processing of HPMCAS 126 G had no negative impact on abiraterone dissolution, it can be concluded that minimal to no degradation of HPMCAS 126 G takes place during KinetiSol processing.

### 3.8. Pharmacokinetic Study in Beagle Dogs

Cyclodextrins are known to enhance the oral bioavailability of drugs by enhancing their solubility. However, when cyclodextrins are used in excess in oral formulations, they can reduce drug absorption due to constant binding with the free drug [67]. Thus, in addition to in vitro analysis, it is imperative to study the effect of KSDs in vivo. So, the polymer-based KSDs along with oligomer-based KSDs in vivo were studied. Moreover, we decided to study binary KSDs along with ternary KSDs to evaluate the impact of the addition of a ternary component.

In order to develop a viable dosage form for abiraterone delivery, the KSDs were compressed into immediate release tablet formulation. Three formulations containing Lot 2 KSD, Lot 5 KSD and Lot 6 KSD were compressed into Lot 2 Tablet, Lot 5 Tablet, and Lot 6 Tablet respectively. Each tablet contained 44.6 mg of abiraterone. All tablets had optimum hardness, assay, purity, acceptable friability, disintegration time, and dissolution. These tablets were tested and compared against generic abiraterone acetate tablets containing 250 mg of abiraterone acetate.

**Figure 9:**
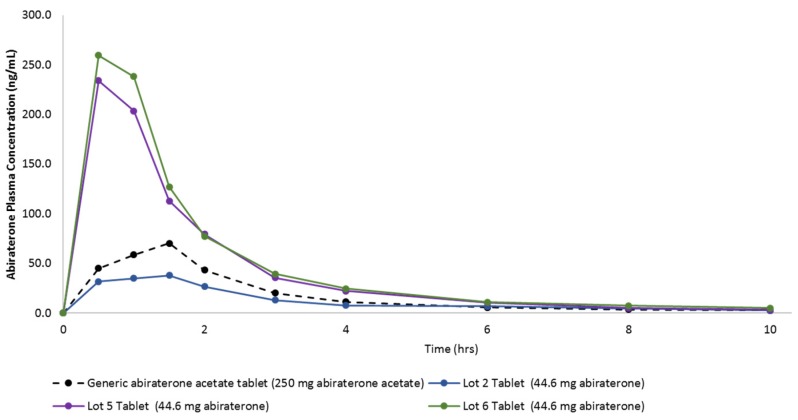
In vivo plasma concentration versus time profiles from oral dosing of the generic abiraterone acetate tablet, the Lot 2 Tablet, the Lot 5 Tablet, and the Lot 6 Tablet in fasted non-naïve male beagle dogs.

Figure 9 shows the in vivo plasma concentration versus time profiles of these tablets, and Table 5 lists the pharmacokinetic parameters. It can be seen that the Lots 5 and 6 Tablets containing HPBCD achieved a higher maximum abiraterone plasma concentration (i.e., C_max_ at one fifth the dose compared to the generic abiraterone acetate tablets). This proves that (a) the Lots 5 and 6 KSDs can deliver high amounts of abiraterone and (b) the concentration of HPBCD in these lots had no negative impact on the abiraterone absorption process.

If we dose adjust Lot 2 Tablet C_max_, then it too is higher than the C_max_ of the generic abiraterone acetate tablets. The time to achieve C_max_ (i.e., the T_max_) of the generic abiraterone acetate tablet and the Lot 2 tablet were comparable, but the T_max_ of the Lots 5 and 6 tablets were lower. This suggests that HPBCD-based tablets can achieve faster abiraterone supersaturation, as observed in the in vitro dissolution study. Although not statistically significant, but polymer-based KSD (i.e., the Lot 2 Tablet) was able to enhance the abiraterone bioavailability by 3.4-fold. The Lots 5 and 6 tablets can enhance abiraterone bioavailability with statistical significance by 12.4-fold (*p* = 0.0210) and 13.8-fold (*p* = 0.0020), respectively.

It is interesting to note that although not statistically significant, the AUC_0–10 h_ of ternary KSD based the Lot 6 Tablet was higher than binary KSD based Lot 5 Tablet, thereby indicating the positive effect of the addition of a ternary component. Also, the Lots 5 and 6 tablets were able to significantly reduce the inter-subject variability (i.e., % CV for C_max_ and AUC_0–10 h_) compared to generic abiraterone acetate tablets. This is because ASDs entail more consistent and complete dissolution of drugs [26]. Overall, the pharmacokinetics of abiraterone were drastically improved by HPBCD-based KSDs.

## 4. Conclusions

From this study, we found that KinetiSol technology enabled the development of abiraterone ASDs that can enhance the dissolution and pharmacokinetics of abiraterone. Also, this study demonstrates for the first time that KinetiSol technology can process both long-chain linear polymers and short-chain cyclic oligomers with low molecular weights. Overall, HPBCD-based KSDs improved the dissolution and pharmacokinetics of abiraterone. These KSDs have a potential to eliminate food effects, enhance abiraterone efficacy, reverse resistance to abiraterone in prostate cancer patients. These formulations should be explored further to investigate their impact on therapeutic outcomes.

## 5. Patents

Urvi Gala, Dave Miller and Robert O. Williams III are coinventors on intellectual property related to this work (US20190091339A1).

## Figures and Tables

**Table 1:** Primary polymers/Oligomers selected for development of binary KSDs of abiraterone [35,45,46,47].

Polymer/Oligomer	Hydroxy Propyl Methyl Cellulose (HPMC E3)	Hydroxy Propyl Methyl Cellulose (HPMC E5)	Polyvinyl Pyrrolidone (PVP 30)	Polyvinyl Acetate Phthalate (PVAP)	Hydroxy Propyl β Cyclodextrin (HPBCD)
Commercial Product used	Methocel™ E3 Premium LV	Methocel™ E5 Premium LV	Kollidon^®^ 30	Phthalavin^®^	Kleptose® HPB
Chemistry	Cellulose based	Cellulose based	Pyrrolidone based	Phthalate based	Glucose based
Monomer Structure	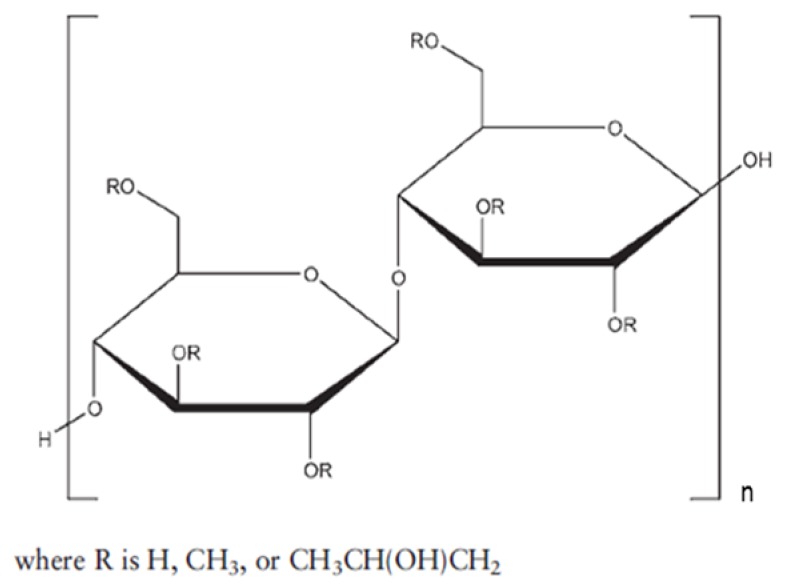		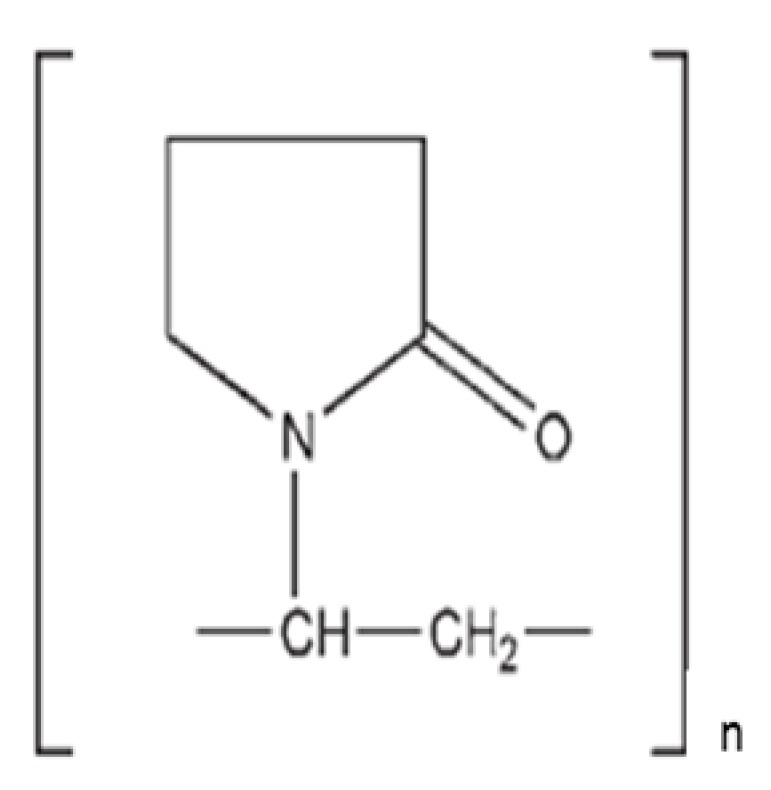	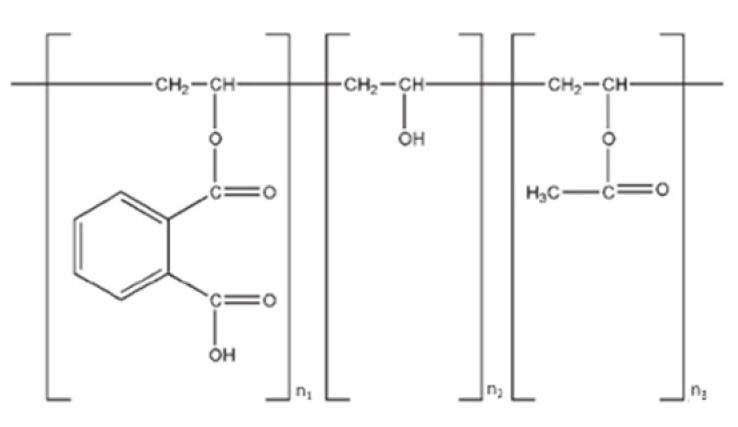	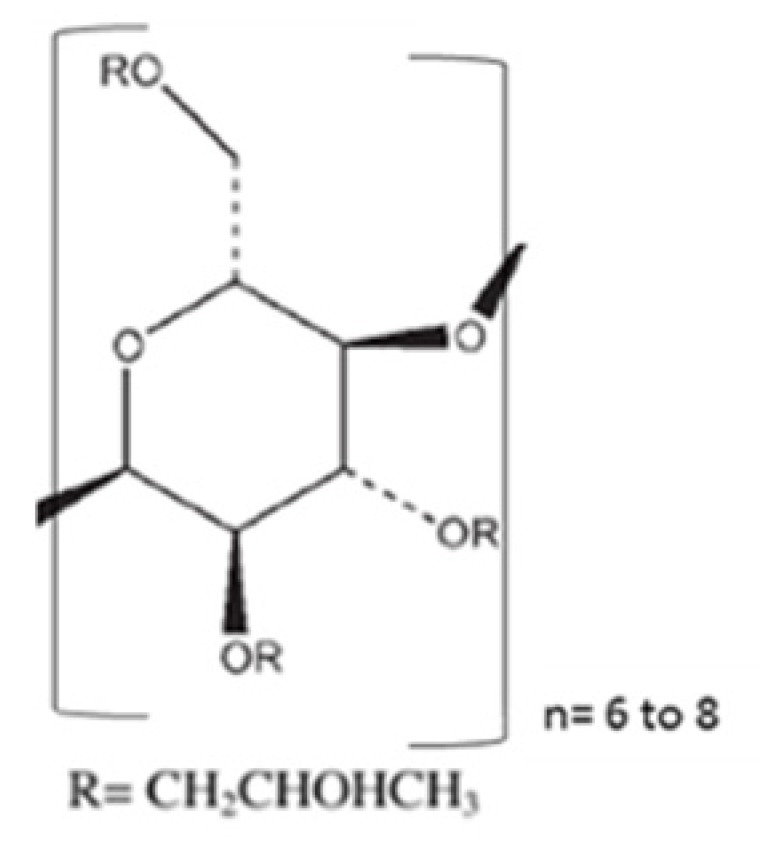
Architecture	Long-chain Linear Polymer	Long-chain Linear Polymer	Long-chain Linear Polymer	Long-chain Linear Polymer	Short-chain Cyclic Oligomer
Molecular Weight	~20,000	~28,700	~50,000	~60,700	1399
Viscosity	2.4–3.6 (mPa.s: 2% *w/v* in water at 20 °C)	4.0–6.0 (mPa.s: 2% *w/v* in water at 20 °C)	5.5–8.5 (mPa.s: 10% *w/v* in water at 20 °C)	7–11 (mPa.s: in water at 25 °C)	<1.5 (mPa.s: 10% *w/v* in water at 25 °C)

**Table 2:** Binary KSD compositions, processing parameters, and their corresponding appearance.

Lot No.	Composition		Batch Size (g)	Processing Temperature (°C)	Shear Stress (Rotational Speed (rpm))	* Processing Time (Seconds)	Appearance
	Drug (% wt)	Primary Polymer/Oligomer (% wt)					
1	Abiraterone (10)	HPMC E3 (90)	10	160	4000, 5000, 6000	10 + 10 + 6.9	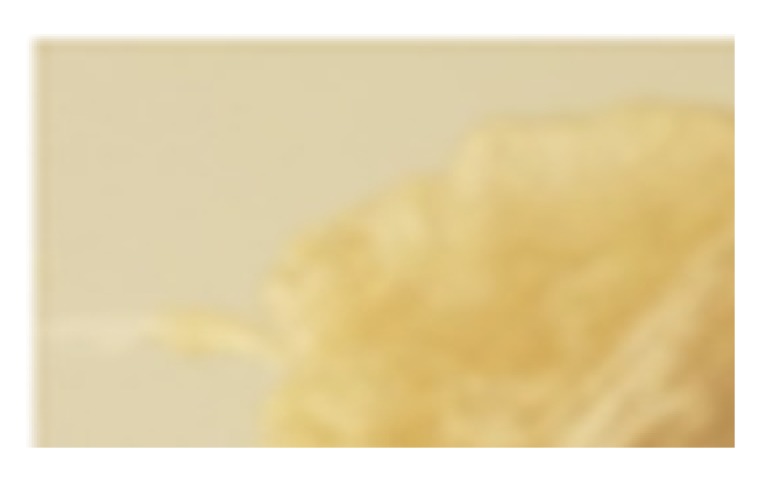
2	Abiraterone (10)	HPMC E5 (90)	10	160	4000, 5000, 6000	10 + 10 + 7.1	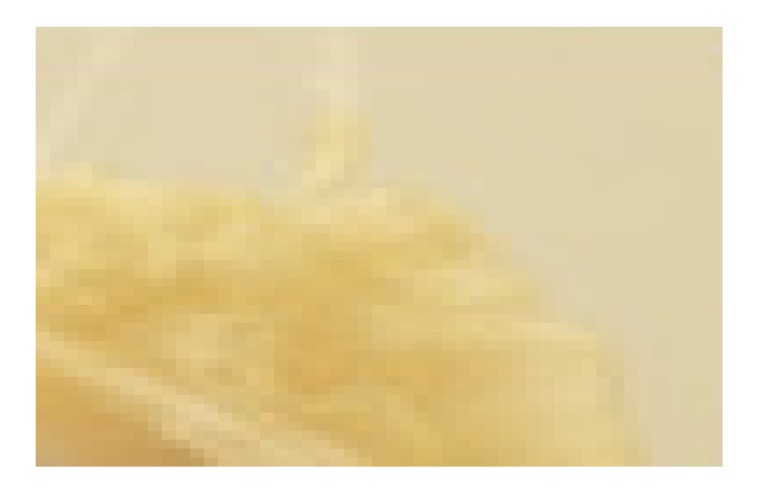
3	Abiraterone (10)	PVP K30 (90)	10	160	4000, 5000, 6000	10 + 10 + 3.7	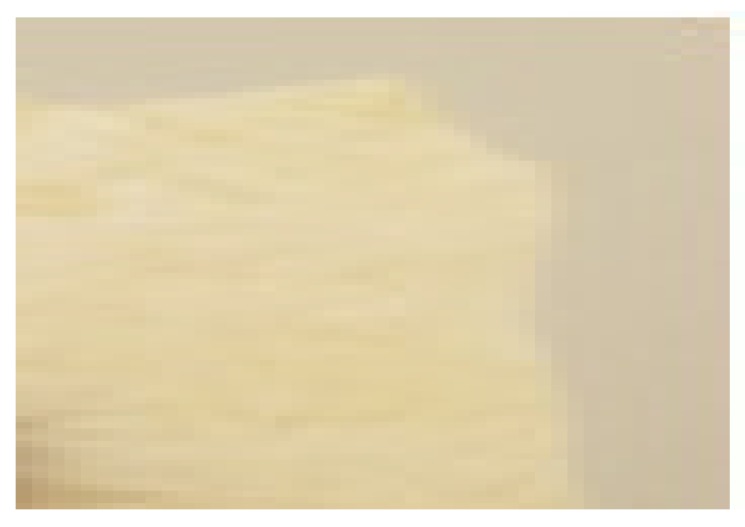
4	Abiraterone (10)	PVAP (90)	10	160	4000, 5000, 6000	10 + 10 +23	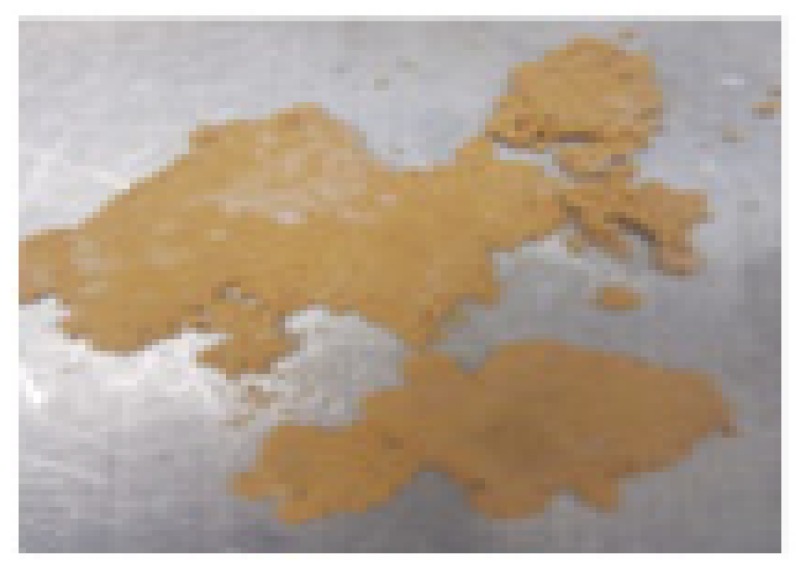
5	Abiraterone (10)	HPBCD (90)	10	160	4000, 5000, 6000	10+10+6.3	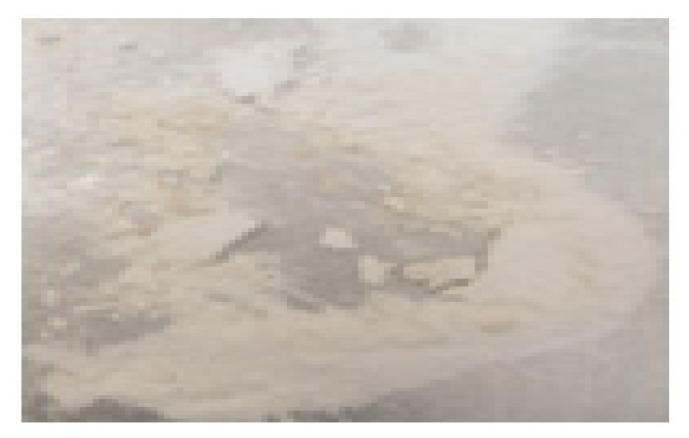

* Each processing time number corresponds to the time spent on the corresponding shear stress stage (i.e., the rotation speed stage).

**Table 3:** Relative area-under-the-drug dissolution curve for the selection of suitable ternary components (i.e., secondary polymers).

Sample	Percent Rel. AUDC_Total_ (Relative to Lot 5 KSD)	Percent Rel. AUDC_0.01 N HCl_ (Relative to Lot 5 KSD)	Percent Rel. AUDC_FaSSIF_ (Relative to Lot 5 KSD)
Lot 5 KSD	100.0	100.0	100.0
Abiraterone API	8.4	1.8	46.2
Generic Abiraterone Acetate Tablet	18.4	3.6	104.1
Lot 5 KSD with HPMC E15	89.0	83.1	109.1
Lot 5 KSD with HPMC E50	89.2	82.1	113.8
Lot 5 KSD with PVP K90	91.8	87.9	105.0
Lot 5 KSD with HPMCAS 126 G	108.9	83.5	244.8
Lot 5 KSD with HPMCAS 716 G	85.9	86.1	77.4
Lot 5 KSD with HPMCAS 912G	88.1	79.7	134.6
Lot 5 KSD with Na CMC	85.4	85.8	79.1
Lot 5 KSD with PVAP	80.4	78.0	85.3
Lot 5 KSD with Eudragit L-100 55	93.8	90.3	107.5

**Table 4:** Ternary KSD composition, processing parameters, and appearance.

Lot No.	Composition			Batch Size (g)	Processing Temperature (°C)	Shear Stress (Rotational Speed-Rpm)	* Processing Time (Seconds)	Appearance
	API (% Wt)	Primary Cyclic Oligomer (% Wt)	Secondary Polymer (% Wt)					
6	Abiraterone (10)	HPBCD (80)	HPMCAS 126 G (10)	10	160	4000, 5000	10 + 3.6	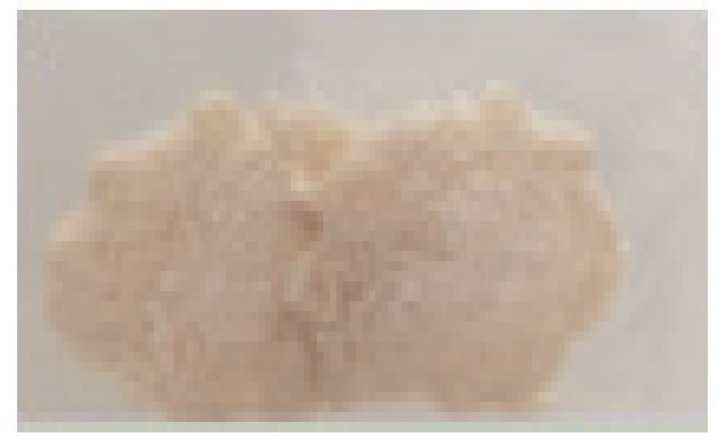
7	Abiraterone (10)	HPBCD (70)	HPMCAS 126 G (20)	10	160	4000, 5000	10 + 6.3	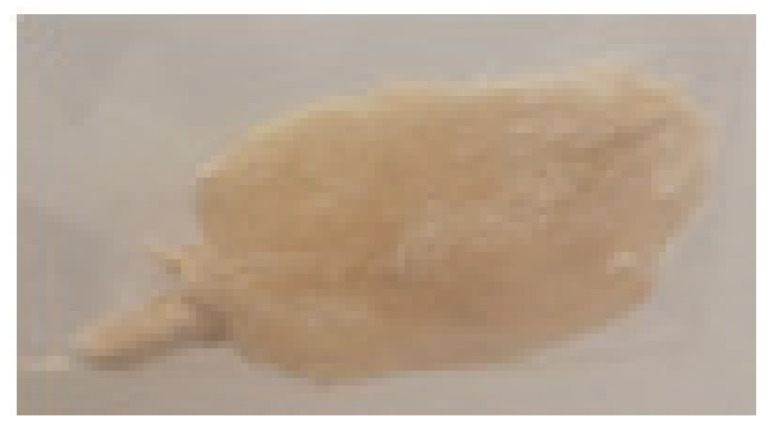
8	Abiraterone (10)	HPBCD (60)	HPMCAS 126 G (30)	10	160	4000, 5000	10 + 5.2	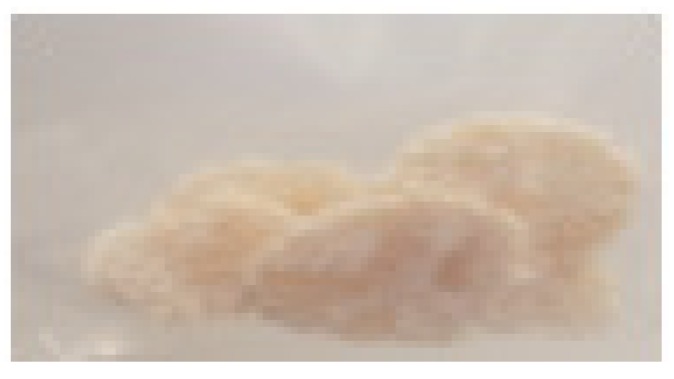
9	Abiraterone (10)	HPBCD (50)	HPMCAS 126 G (40)	10	160	4000	9.6	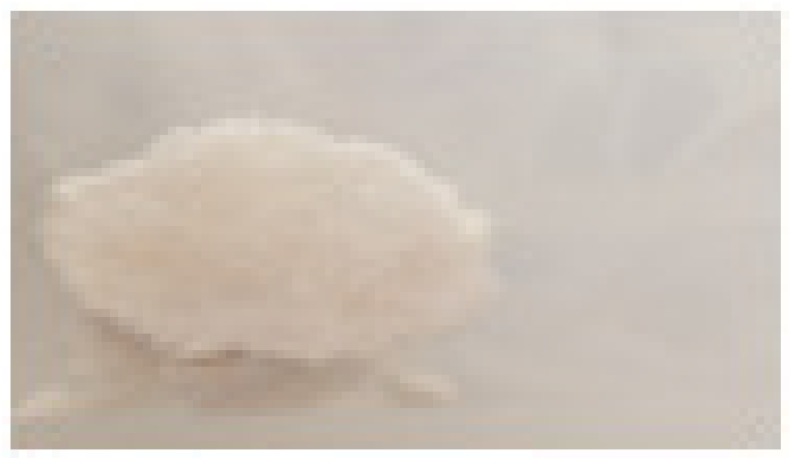

* Each processing time number corresponds to the time spent on the corresponding shear stress stage (i.e., the rotation speed stage.

**Table 5:** Results from the in vivo pharmacokinetic (PK) study in male beagle dogs.

		Generic Abiraterone Acetate Tablet (250 mg Abiraterone Acetate)		Lot 2 Tablet (44.6 mg Abiraterone)		Lot 5 Tablet (44.6 mg Abiraterone)		Lot 6 Tablet (44.6 mg Abiraterone)	
PK parameters	Units	Average	%CV	Average	%CV	Average	%CV	Average	%CV
C_max_	ng/mL	86.32	66.57%	53.58	58.84%	280.00	33.51%	305.00	20.72%
T_max_	hr	1.20	37.27%	1.20	55.90%	0.80	34.23%	0.70	39.12%
T_1/2_	hr	4.78	50.70%	3.12	42.20%	2.30	32.30%	3.47	28.90%
AUC_0–10 h_	ng*hr/mL	177.29	77.53%	122.21	25.64%	438.01	34.29%	487.58	14.11%
F Value (Dose Adjusted)	unitless	1.0		3.4		12.4		13.8	
